# Author Correction: Genetic instability from a single S phase after whole-genome duplication

**DOI:** 10.1038/s41586-022-05099-w

**Published:** 2022-07-21

**Authors:** Simon Gemble, René Wardenaar, Kristina Keuper, Nishit Srivastava, Maddalena Nano, Anne-Sophie Macé, Andréa E. Tijhuis, Sara Vanessa Bernhard, Diana C. J. Spierings, Anthony Simon, Oumou Goundiam, Helfrid Hochegger, Matthieu Piel, Floris Foijer, Zuzana Storchová, Renata Basto

**Affiliations:** 1 Institut Curie, PSL Research University, CNRS, UMR144, Biology of Centrosomes and Genetic Instability Laboratory, Paris, France; 2 European Research Institute for the Biology of Ageing, University of Groningen, University Medical Center Groningen, Groningen, The Netherlands; 3 Department of Molecular Genetics, TU Kaiserslautern, Kaiserslautern, Germany; 4 Institut Curie and Institut Pierre Gilles de Gennes, PSL Research University, CNRS, UMR 144, Systems Biology of Cell Polarity and Cell Division, Paris, France; 5 Cell and Tissue Imaging Facility (PICT-IBiSA), Institut Curie, PSL Research University, Centre National de la Recherche Scientifique, Paris, France; 6 Genome Damage and Stability Centre, School of Life Sciences, University of Sussex, Brighton, UK; 7 Present Address: Molecular, Cellular, and Developmental Biology Department, University of California, Santa Barbara, CA USA

**Keywords:** Genomic instability, Cell growth, Polyploidy

Correction to: *Nature* 10.1038/s41586-022-04578-4Published online 30 March 2022

In the version of this article initially published, the third sentence of the abstract, now reading “This is a key question because single whole-genome duplication events such as cytokinesis failure can promote tumorigenesis^9^ and DNA double-strand breaks^10^” was truncated and did not mention double-strand breaks or cite ref. 10 (Pedersen, R. S. et al. Profiling DNA damage response following mitotic perturbations. *Nat. Commun.* **7**, 13887 (2016)). The reference has now been included in the HTML and PDF versions of the article

