# Supplementary material for: Genetic instability from a single S phase after whole-genome duplication
Source: Nature. 2022 Mar 30;604(7904):146–51. doi: 10.1038/s41586-022-04578-4 (PMC8986533; doi:10.1038/s41586-022-04578-4)
Supplement: Supplementary file 2 — Reporting Summary [file 41586_2022_4578_MOESM2_ESM.pdf]

nature portfolio

Corresponding author(s): Renata Basto
Simon Gemble

Last updated by author(s): Feb 4, 2022

Reporting Summary

Nature Portfolio wishes to improve the reproducibility of the work that we publish. This form provides structure for consistency and transparency in reporting. For further information on Nature Portfolio policies, see our Editorial Policies and the Editorial Policy Checklist.

Statistics

For all statistical analyses, confirm that the following items are present in the figure legend, table legend, main text, or Methods section.

n/a	Confirmed	
☐	☒	The exact sample size (*n*) for each experimental group/condition, given as a discrete number and unit of measurement
☐	☒	A statement on whether measurements were taken from distinct samples or whether the same sample was measured repeatedly
☐	☒	The statistical test(s) used AND whether they are one- or two-sided<br>*Only common tests should be described solely by name; describe more complex techniques in the Methods section.*
☒	☐	A description of all covariates tested
☒	☐	A description of any assumptions or corrections, such as tests of normality and adjustment for multiple comparisons
☐	☒	A full description of the statistical parameters including central tendency (e.g. means) or other basic estimates (e.g. regression coefficient) AND variation (e.g. standard deviation) or associated estimates of uncertainty (e.g. confidence intervals)
☐	☒	For null hypothesis testing, the test statistic (e.g. *F*, *t*, *r*) with confidence intervals, effect sizes, degrees of freedom and *P* value noted<br>*Give P values as exact values whenever suitable.*
☒	☐	For Bayesian analysis, information on the choice of priors and Markov chain Monte Carlo settings
☒	☐	For hierarchical and complex designs, identification of the appropriate level for tests and full reporting of outcomes
☐	☒	Estimates of effect sizes (e.g. Cohen's *d*, Pearson's *r*), indicating how they were calculated

Our web collection on statistics for biologists contains articles on many of the points above.

Software and code

Policy information about availability of computer code

Data collection	Flow cytometry acquisitions were performed using BD FACSDiva Software Version 8.0.1<br>Microscopy acquisitions were performed using Metamorph 7.10.1 software (Molecular Devices, USA)
Data analysis	Image J software V2.1.0/1.53c was used to analyze most of the data. Custom made plugins were used to quantify DNA damage in cells and in tissues. After manual segmentation of the nuclei, a thresholding operation was used to determine the percentage of gamma H2Av positive pixels (coverage) and their average intensity in a single z plane in the center of the nucleus. Coverage and intensity were multiplied to obtain the gamma H2Av. For human cells gamma H2AX signals were measured using z-projection stacks after thresholding. Both FI and the percentage of nuclear coverage was obtained for each nucleus. Gamma H2AX index was obtained multiplying FI by the coverage. Statistical tests were performed using GraphPad Prism version 7.00 for Mac, GraphPad Software. Quantitative 4D live imaging of endogenous DNA replication, 3D reconstruction and analysis were done using Imaris Software v.9.6.0. Flow cytometry data were analyzed using FlowJo software 10.6.0

For manuscripts utilizing custom algorithms or software that are central to the research but not yet described in published literature, software must be made available to editors and reviewers. We strongly encourage code deposition in a community repository (e.g. GitHub). See the Nature Portfolio guidelines for submitting code & software for further information.

Data

Policy information about availability of data

All manuscripts must include a data availability statement. This statement should provide the following information, where applicable:

- Accession codes, unique identifiers, or web links for publicly available datasets
- A description of any restrictions on data availability
- For clinical datasets or third party data, please ensure that the statement adheres to our policy

The datasets generated during and/or analysed during the current study are available from the corresponding author.

Field-specific reporting

Please select the one below that is the best fit for your research. If you are not sure, read the appropriate sections before making your selection.

☒ Life sciences ☐ Behavioural & social sciences ☐ Ecological, evolutionary & environmental sciences

For a reference copy of the document with all sections, see nature.com/documents/nr-reporting-summary-flat.pdf

Life sciences study design

All studies must disclose on these points even when the disclosure is negative.

Sample size	At least 100 interphase cells were analyzed to determine DNA damage levels. The size of the sample was chosen to offer sufficient statistical power.
Data exclusions	We did not exclude any data.
Replication	All experiments were considered as replicates.
Randomization	Randomization was not relevant in this study.
Blinding	We tried to analyze DNA damage and quantify the phenotypes and behaviors described in this article in a blind manner initially. However, this was not possible as tetraploid cells can be easily distinguished from diploid cells. In any case, in terms of immunostaining experiments, the distinction between both cell types was solely based on the characteristics mentioned in the paper- cell and nuclear size and centrosome number.

Reporting for specific materials, systems and methods

We require information from authors about some types of materials, experimental systems and methods used in many studies. Here, indicate whether each material, system or method listed is relevant to your study. If you are not sure if a list item applies to your research, read the appropriate section before selecting a response.

Materials & experimental systems

n/a	Involved in the study	
☐	☒	Antibodies
☐	☒	Eukaryotic cell lines
☒	☐	Palaeontology and archaeology
☐	☒	Animals and other organisms
☒	☐	Human research participants
☒	☐	Clinical data
☒	☐	Dual use research of concern

Methods

n/a	Involved in the study	
☒	☐	ChIP-seq
☐	☒	Flow cytometry
☒	☐	MRI-based neuroimaging

Antibodies

Antibodies used	For Immunofluorescence:<br>Primary and secondary antibodies were used at the following concentrations: Guinea pig anti CEP192 antibody (1/500; Basto lab)60, rabbit anti-beta catenin (1/250; C2206 from Sigma-Aldrich, RRID:AB_476831), mouse anti-gamma H2A.X phospho S139 (1/1000; ab22551 from Abcam, RRID:AB_447150), mouse anti-XRCC1 (1/500; ab1838 from Abcam, RRID:AB_302636), rabbit anti-Rad51 (1/500; ab133534 from Abcam, RRID:AB_2722613), mouse anti-KU80 (1/200; MA5-12933 from ThermoFisher, RRID:AB_10983840), rabbit anti-FANCD2 (1/150; NB100-182SS from Novusbio, RRID:AB_1108397), mouse anti-53BP1 (1/250; MAB3802 from Millipore, RRID:AB_2206767), rabbit anti-H2Av (1/500; 600-401-914 from Rockland; RRID: AB_11183655), Alexa Fluor® 647 Phalloidin (1/250; A22287 from ThermoFisher Scientific, RRID:AB_2620155), goat anti-Rabbit IgG (H+L) Highly Cross-Adsorbed Secondary Antibody, Alexa Fluor 647 (1/250; A21245 from ThermoFisher, RRID:AB_2535813), Goat anti-Guinea Pig IgG (H+L) Highly Cross-Adsorbed

Secondary Antibody, Alexa Fluor 488 (1/250; A11073 from ThermoFisher, RRID:AB_253411), Goat anti-Mouse IgG (H+L) Cross-Adsorbed Secondary Antibody, Alexa Fluor 546 (1/250, A11003 from ThermoFisher, RRID:AB_2534071), Goat anti-Rabbit IgG (H+L) Highly Cross-Adsorbed Secondary Antibody, Alexa Fluor 546 (1/250; A-11035 from Thermo Fisher Scientific, RRID:AB_2534093).

For Western Blot:
- Primary and secondary antibodies were used at the following concentrations
Mouse anti-tubulin (1/5000; T9026 from Sigma, RRID:AB_477593), mouse anti- CDC45 (1/100; sc-55569 from Santa Cruz Biotechnology, RRID:AB_831146), rabbit anti-PCNA (1/500; sc56 from Santa Cruz, RRID:AB_628110), rabbit anti-Actin (1/2000; A5060 from Sigma-Aldrich, RRID:AB_476738), mouse anti-H2B (1/1000; sc-515808 from Santa Cruz Biotechnology), mouse anti-ORC1 (1/100; sc-398734 from Santa Cruz Biotechnology), mouse anti-MCM2 (1/500; 610701 from BD Biosciences, RRID:AB_398024), mouse anti-E2F1 (1/2000; sc251 from Santa Cruz, RRID:AB_627476), mouse anti-CDC6 (1/500; sc-9964 from Santa Cruz, RRID:AB_627236), rabbit anti-CDT1 (1/500; 8064S from Cell Signaling, RRID:AB_10896851), rabbit anti-Treslin (1/500; A303-472A from Betyl, RRID:AB_10953949), Goat anti-Rabbit IgG (H+L) Cross-Adsorbed Secondary Antibody, HRP (1/2500; G21234 from ThermoFisher, RRID:AB_2536530), Peroxidase AffiniPure Goat Anti-Mouse IgG (H+L) (1/2500; 115-035-003 from Jackson ImmunoResearch, RRID:AB_10015289).

For DNA combing:
Antibodies were used at the following concentrations:
Rabbit anti ssDNA (1/5; 18731 from IBL International, RRID:AB_494649), Rat anti CldU (1/10; Ab6326 from Abcam, RRID:AB_2313786), Mouse anti IdU (1/10; 555627 from BD Biosciences, RRID:AB_10015222), mouse Alexa Fluor 647 Donkey (1/25; JIM-715-605-151 from Biozol), Rat Alexa Fluor 594 Donkey (1/25; JIM-712-585-153 from Biozol), Rabbit Brilliant Violet 480 Donkey (1/25; 711-685-152 from Jakcson Immuno Research, RRID:AB_2651109).

Validation

Commercial antibodies were initially considered using the website information and by testing conditions that should increase or decrease the signals expected. For example- anti-gamma H2A.X phospho S139 signals should be increased upon the generation of DNA damage with external agents such as Aphidicoline, which we used. The single non- commercial antibody used was CEP192 antibody (1/500; Basto lab) (Vargas et al., 2019, Current Biology, PMID: 31495584; previously characterised with CEP192 depletion and by western blot on the size of the expected band). All secondary antibodies have been tested on multiple occasions in different projects from the lab. Different combinations of secondary antibodies have already been tested.

Eukaryotic cell lines

Policy information about cell lines

Cell line source(s)

hTERT RPE-1 cells (ATCC Cat# CRL-4000, RRID:CVCL_4388; HEK293 cells (ATCC Cat# CRL-1573, RRID:CVCL_0045); BJ cells (ATCC Cat# CRL-4001, RRID:CVCL_6573) and HCT116 cells (ATCC Cat# CCL-247, RRID:CVCL_0291)

Authentication

Cells were authenticated using the Institut Curie genotype validation.

Mycoplasma contamination

All cells were routinely tested for Mycoplasm contamination. All cells used in this paper were mycoplasm-free.

Commonly misidentified lines
(See ICLAC register)

We did not use any mis-identified cell line.

Animals and other organisms

Policy information about studies involving animals; ARRIVE guidelines recommended for reporting animal research

Laboratory animals

Drosophila melanogaster- w background.
Mutants analyzed were L3 male with matched controls.
UAS E2F1 OE: M{UAS-E2f1.ORF}ZH-86Fb
sqh mutant: y w sqh1/FM7
sqh mutant + wrnGal4: y w sqh1/FM7;WornGal4/Cyo
PAV RNAi: y1 v1; P{TRiP.HMJ02232}attP40

Wild animals

This study did not involved wild animals.

Field-collected samples

This study did not involved samples collected in the field.

Ethics oversight

Ethical approval is not required for work with Drosophila melanogaster (invertebrate animal- fruit fly). European research guidelines in terms of disposal and transgenic reporting were followed.

Note that full information on the approval of the study protocol must also be provided in the manuscript.

Flow Cytometry

Plots

Confirm that:

☒ The axis labels state the marker and fluorochrome used (e.g. CD4-FITC).

☒ The axis scales are clearly visible. Include numbers along axes only for bottom left plot of group (a 'group' is an analysis of identical markers).

☒ All plots are contour plots with outliers or pseudocolor plots.

☒ A numerical value for number of cells or percentage (with statistics) is provided.

Methodology

Sample preparation	A mix of diploid and tetraploid cells (see "generation of tetraploid cells" section) were incubated with 2µg/ml Hoescht (94403 from Sigma Aldrich) for 1 hour at 37°C, 5% CO2. Then, a single cell suspension was generated. Cells were washed using PBS 1X, the supernatant was removed and cells were resuspended in cold cell culture medium at 1x107 cell per ml and kept at 4° C during all the experiment. FACS sorting was performed using Sony SH800 FACS (BD FACSDiva Software Version 8.0.1).
Instrument	Sony SH800 and BD LSRII
Software	BD FACSDiva Software Version 8.0.1
Cell population abundance	Post-sort analysis was performed to determine the purity of the sorted populations (see extended data Fig 8d-e)
Gating strategy	Compensation was performed using the appropriate negative control samples. Experimental samples were then recorded and sorted using gating tools to select the populations of interest. RFP+ / GFP- negative cells (G1 cells) were first selected. Then, in this population, DNA content was used to segregate diploid (2n) and tetraploid (4n) G1 cells. Once gates have been determined, diploid and tetraploid G1 cells were sorted into external collection tubes.

☒ Tick this box to confirm that a figure exemplifying the gating strategy is provided in the Supplementary Information.
